# Diagnosis of Kallmann Syndrome in a Young Adult Male

**DOI:** 10.7759/cureus.65916

**Published:** 2024-08-01

**Authors:** Kushal Bothara, Purnachandra Lamghare, Eshan Chetan Durgi, Karishma Krishnani

**Affiliations:** 1 Radiodiagnosis, Dr. D. Y. Patil Medical College, Hospital and Research Centre, Dr. D. Y. Patil Vidyapeeth, Pune, IND; 2 Radiodiagnosis, Dr. D. Y. Patil Medical College, Hospital and Research Centre, Dr. D. Y. Patil Vidyapeeth, Pune, IND, Pune, IND; 3 Radiodiagnosis, Dr. D. Y. Patil Medical College, Hospital and Research Centre, Dr. D.Y. Patil Vidyapeeth,Pimpri, Pune, IND

**Keywords:** hormonal assay, anosmia, genetic disorder, hypoplastic olfactory bulb, gnrh, hypogondadotropic hypogonadism

## Abstract

Kallmann syndrome is an anosmic variety of GnRH (gonadotropin-releasing hormone) deficiency. A young adult male with anosmia since childhood presented with features of failed sexual maturation and underwent an ultrasound of the genital system and an MRI (magnetic resonance imaging) of the brain. MRI revealed absent olfactory bulbs in bilateral olfactory grooves with hypoplastic olfactory sulci on both sides. Ultrasound showed atrophic testes, while the anterior pituitary appeared normal on MRI. The clinical, hormonal, and imaging findings were characteristic of Kallmann syndrome. MRI serves as a crucial tool in its diagnosis.

## Introduction

Kallmann syndrome is an anosmic variety of (gonadotropin-releasing hormone) GnRH deficiency [1]. It usually results from mutations involving one or more genes involved in olfactory bulb morphogenesis and the subsequent migration of GnRH neurons from their site of origin to their final location. It is an X-linked disorder, more common in males [2], resulting in GnRH deficiency. Diagnosis is based on a combination of clinical findings, hormonal evaluation, and imaging findings.

## Case presentation

A 20-year-old male presented with complaints of failed sexual maturation and anosmia since childhood. He also reported decreased hearing in his left ear. No other associated medical issues were noted, and there was no history of similar complaints in his family. The patient has two siblings - a brother and a sister - with normal sexual maturity. Upon arrival at our hospital, the patient underwent an MRI of the brain, an ultrasound of the abdomen and pelvis, and hormonal assays.

**Figure 1:**
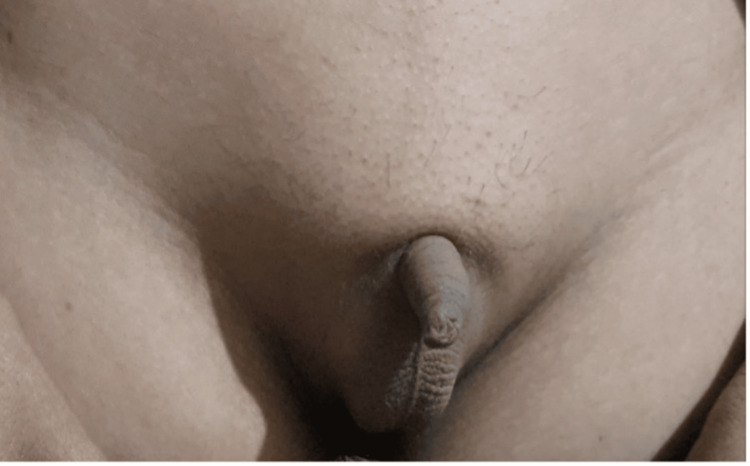
The clinical image demonstrating micro-penis.

Clinical examination initially revealed hormonal abnormalities, including absent secondary sexual characteristics, micropenis, a high-pitched voice, and decreased pubic, axillary, and facial hair (Figure 1).

**Figure 2:**
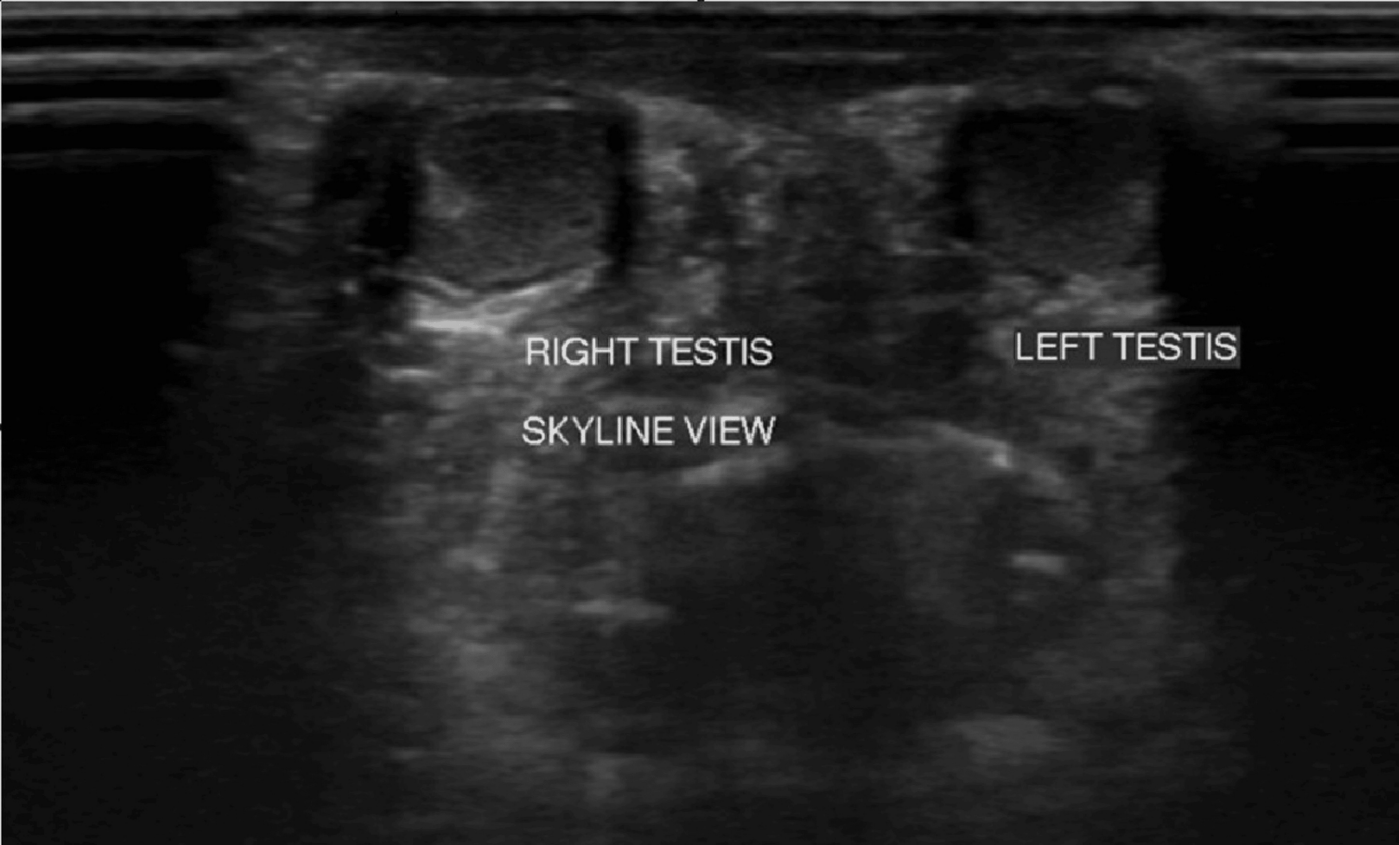
Ultrasound image showing skyline view showing both the atrophic testes.

On ultrasonography, normal functioning bilateral kidneys were observed, with a renal calculus noted in the right kidney. In inguino-scrotal ultrasonography, both testes appeared smaller in size, with the right testis measuring approximately 0.32 cc and the left testis measuring approximately 0.27 cc. Normal testis volume typically ranges from 12 to 30 cc. Therefore, in this case, the findings suggested testicular atrophy. Importantly, both testes were observed within the scrotal sac, ruling out cryptorchidism (Figure 2).

**Figure 3:**
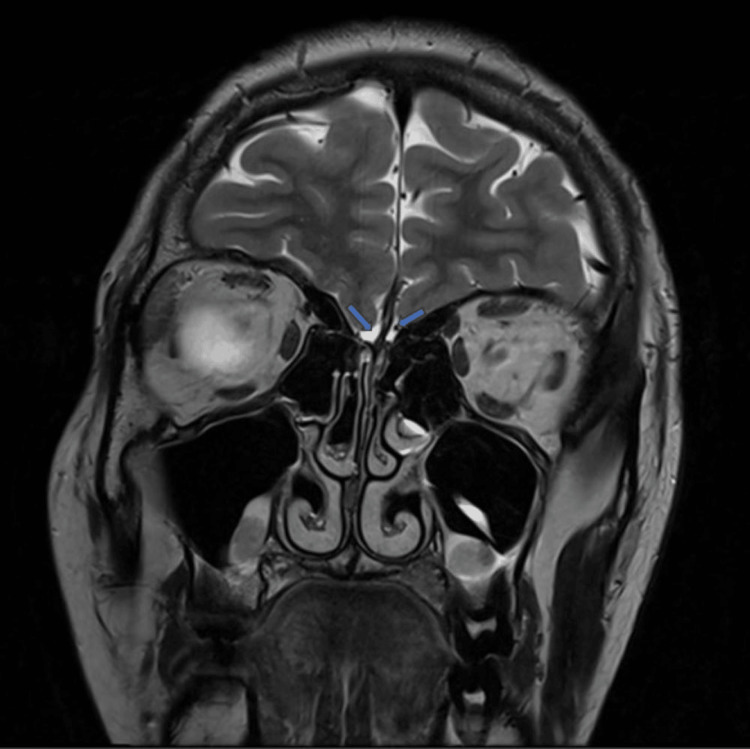
Coronal T2-weighted MRI shows absent olfactory bulbs in bilateral olfactory grooves with hypoplastic olfactory sulci on both sides (blue arrows).

**Figure 4:**
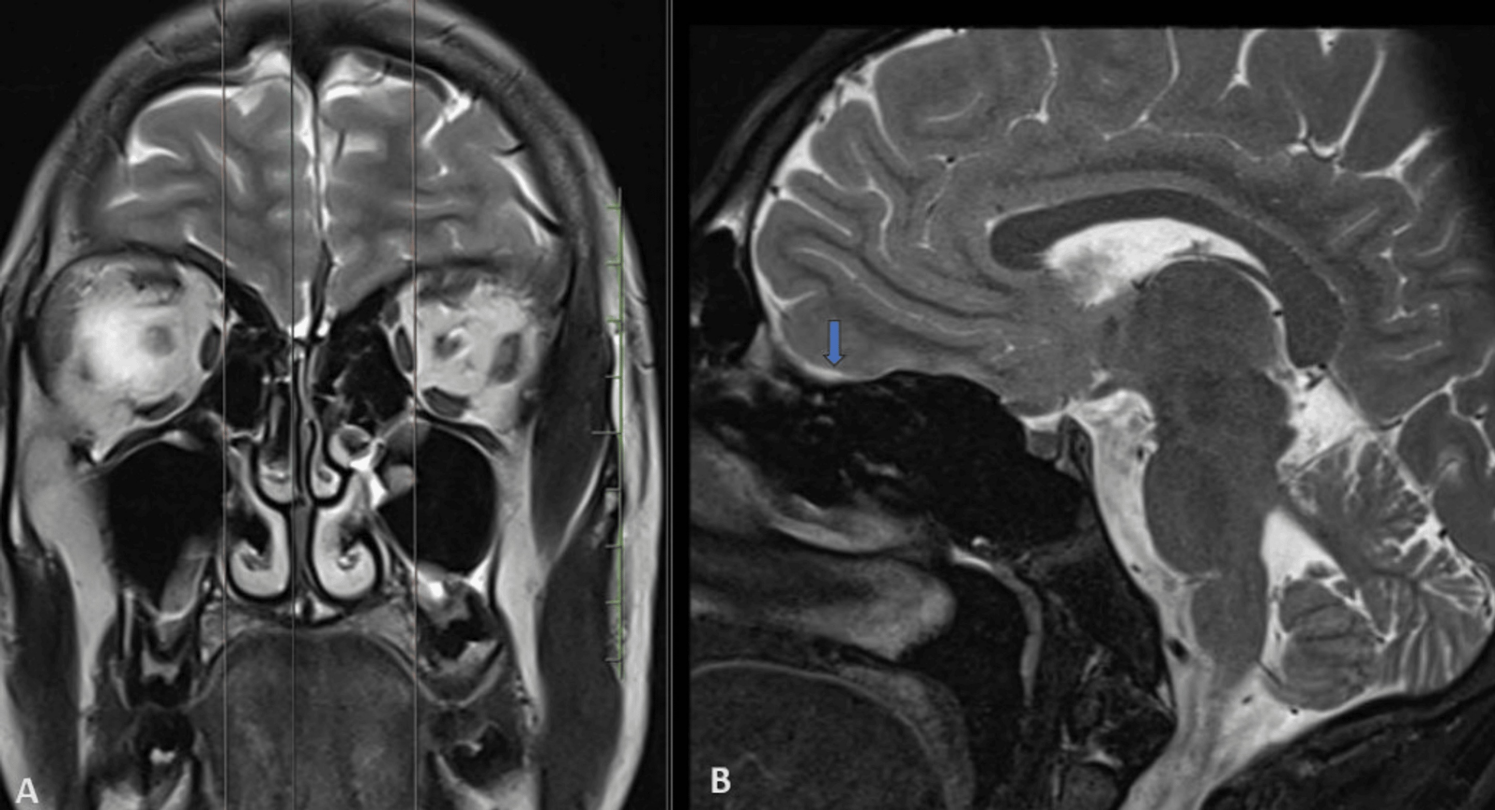
T2-weighted MRI in coronal section (A) with sagittal section (B) at corresponding level show absent olfactory bulb in right olfactory groove with hypoplastic olfactory sulcus (blue arrow).

**Figure 5:**
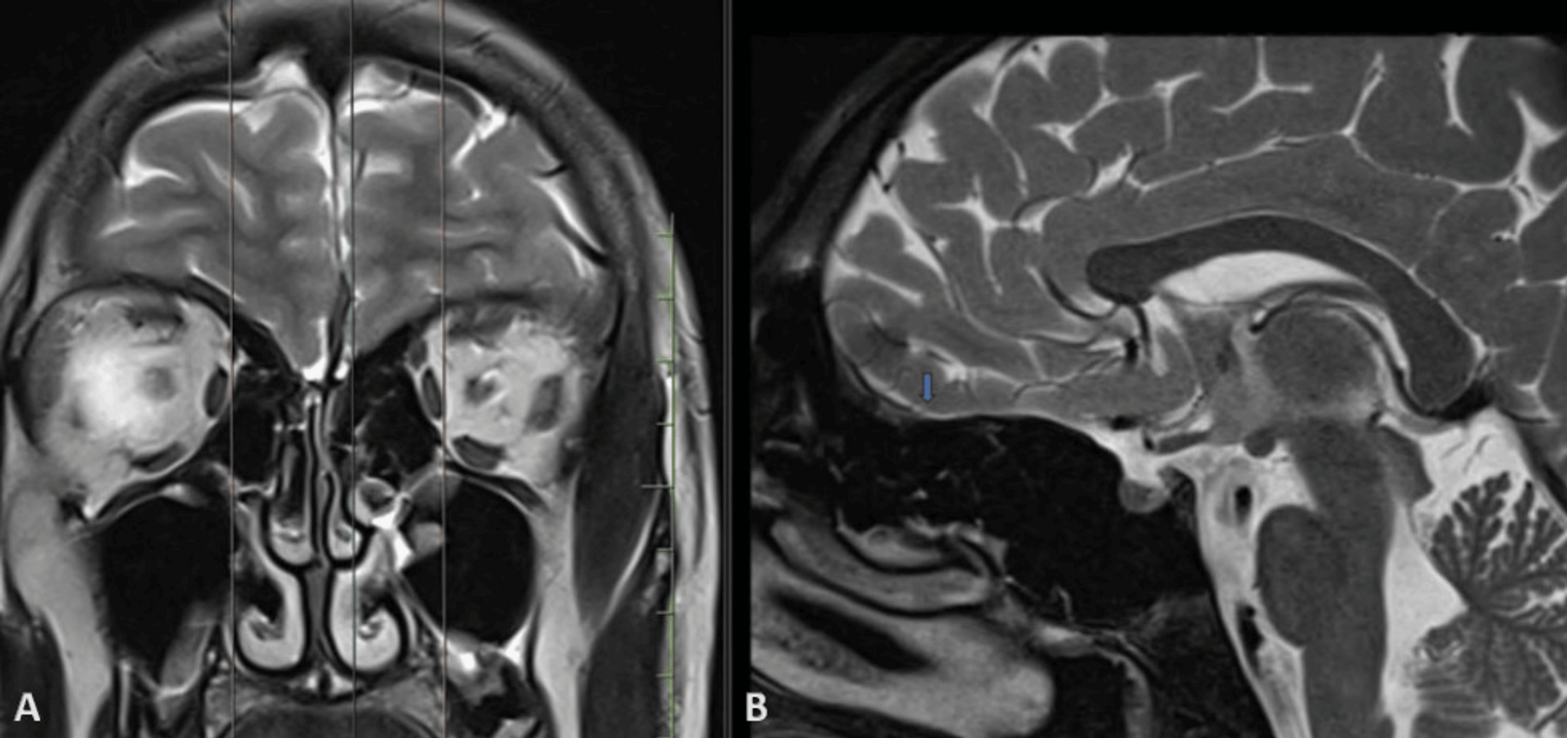
Coronal T2-weighted MRI in coronal (A) section with sagittal section (B) at corresponding level show absent olfactory bulb in left olfactory groove with hypoplastic olfactory sulcus (blue arrow).

**Figure 6:**
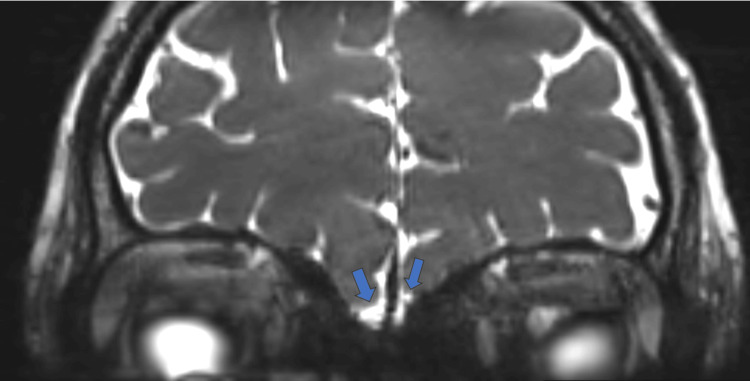
CISS (constructive interference in steady state) MRI sequence showing absent olfactory bulbs (blue arrows).

The patient underwent an MRI, which revealed the absence of olfactory bulbs in bilateral olfactory grooves and bilateral hypoplastic olfactory sulci (Figures 3-6).

With these examination findings, the patient underwent further hormonal assays. Table 1 displays the results of the hormonal assay, indicating reduced levels of follicle-stimulating hormone (FSH), luteinizing hormone (LH), and testosterone compared to the reference ranges.

**Table 1:** Hormonal assay results. FSH: follicle-stimulating hormone, LH: luteinizing hormone.

Test	Observed value	Reference range
FSH	0.46 mIU/ml	1.40–15.40 mIU/ml
LH	0.10 mIU/ml	0.56–12.07 mIU/ml
Testosterone level	9.76 ng/dl	300–950 ng/dl

Based on the above findings, the patient was diagnosed with Kallmann syndrome. Hormonal replacement therapy was initiated, along with the supplementation of vitamin D and bisphosphonates for lifelong management. Surgical intervention was not required. Interval follow-ups with hormonal assays were recommended for monitoring.

## Discussion

Congenital GnRH deficiency can be anosmic or normosmic [3]. Kallmann syndrome is an anosmic variety of GnRH deficiency [1]. It usually results from mutations involving one or more genes involved in olfactory bulb morphogenesis and the subsequent migration of GnRH neurons from their site of origin (in the olfactory placode) into their final location (the pre-optic region of the hypothalamus) [1].

This leads to a deficiency of GnRH, with a resultant deficiency of LH and FSH. LH and FSH are hormones that stimulate the testes, and a reduction in their secretion results in secondary hypogonadism. Hence, this leads to a setting where there is a deficiency of LH, FSH and testosterone.

Kallmann syndrome is known to be associated with mutations in KAL1, genes involved in fibroblast growth factor (FGF) signaling, NELF, genes involved in PROK signaling, WDR11, SEMA3, HS6ST1, CHD7, and FEZF1 [3].

Kallmann syndrome usually involves hypogonadotropic hypogonadism alongside a total lack of the ability to smell (anosmia) [4]. In the present case, the patient has complained of anosmia since childhood. When anosmia is not present, it is commonly known as idiopathic hypogonadotropic hypogonadism (IHH).

Zaghouani et al. [2], in their study of five patients with Kallmann syndrome, reported that all patients had absent olfactory bulbs, with three of them showing absent olfactory sulci and two of them having hypoplastic olfactory sulci. Our case also had absent olfactory bulbs with bilateral hypoplastic olfactory sulci (Figure 3).

Koenigkam-Santos et al. [5] reported that patients with Kallmann syndrome frequently had findings of olfactory bulbs and sulcus aplasia. Specifically, findings of bulb aplasia on MRI in the clinical setting of aplasia were highlighted, which aligns with our own observations in this case. Sometimes, a hypoplastic anterior pituitary may also be seen; however, in the present case, the anterior pituitary was normal.

MRI is the investigation of choice for evaluating olfactory bulb aplasia. The widespread use of MRI in modern medicine has significantly enhanced the radiological assessment of olfactory disorders. The groundbreaking studies by Yousem et al. [6] showcased MRI's capability to provide precise volumetric measurements of the olfactory bulb across different pathological conditions. It allows for detailed imaging of the olfactory bulb and tract, making it highly effective at detecting even subtle damage to the central projection areas involved in the sense of smell.

For the best visualization of the olfactory bulbs, a coronal scan with a large matrix size and minimized intersection gap is advised. This imaging plane is particularly suited for comprehensive anatomical assessment of the olfactory tract, detection of parenchymal lesions, and accurate volumetric measurement of the olfactory bulbs [7]. Axial MRI images allow for the visualization of the olfactory sulci in the frontal lobes; however, they are not ideal for assessing the olfactory bulbs or tracts, which are more clearly seen in coronal views [8].

Kallmann syndrome is known to be associated with renal anomalies, cleft lip/palate, tooth anomalies, pigmentation, cranio-facial anomalies, oculomotor/visuospatial defects, and gut malformations [3]. In the present case, there was no evidence of any cranial or extracranial anomalies.

Kallmann syndrome is usually suspected based on clinical features (delayed puberty) and confirmed using hormonal and imaging findings, such as decreased levels of GnRH, LH, FSH, and testosterone and absence or hypoplasia of the olfactory bulb and sulci.

## Conclusions

Kallmann syndrome is initially suspected based on clinical features such as delayed puberty and is confirmed using hormonal assays and radiological investigations (MRI). Ultrasound is crucial for ruling out associated anomalies, which include renal anomalies and cryptorchidism, and detecting testicular atrophy, as observed in the present case. MRI plays a pivotal role in assessing the olfactory bulbs and ruling out acquired causes of hypogonadism, such as sellar masses or infiltrative diseases of the hypothalamus or pituitary gland. Therefore, MRI plays a significant role in both diagnosing and managing rare genetic disorders like Kallmann syndrome.

## References

[1] Vogl TJ, Stemmler J, Heye B (1994). Kallman syndrome versus idiopathic hypogonadotropic hypogonadism at MR imaging. Radiology.

[2] Zaghouani H, Slim I, Zina NB, Mallat N, Tajouri H, Kraiem C (2013). Kallmann syndrome: MRI findings. Indian J Endocrinol Metab.

[3] Achermann JC, Jameson J (2018). Disorders of sex development. Harrison’s Principles of Internal Medicine.

[4] Dodé C, Hardelin JP (2009). Kallmann syndrome. Eur J Hum Genet.

[5] Koenigkam-Santos M, Santos AC, Versiani BR, Diniz PR, Junior JE, de Castro M (2011). Quantitative magnetic resonance imaging evaluation of the olfactory system in Kallmann syndrome: correlation with a clinical smell test. Neuroendocrinology.

[6] Yousem DM, Turner WJ, Li C, Snyder PJ, Doty RL (1993). Kallmann syndrome: MR evaluation of olfactory system. AJNR.

[7] Knorr JR, Ragland RL, Brown RS, Gelber N (1993). Kallmann syndrome: MR findings. AJNR.

[8] de m Freitas P, Carvalho S, Ribeiro F, Marnoto D, Martins F (2001). Neuroradiology of Kallmann's syndrome. Acta Med Port.

